# Distinct HIV-1 Population Structure across Meningeal and Peripheral T Cells and Macrophage Lineage Cells

**DOI:** 10.1128/spectrum.02508-22

**Published:** 2022-09-29

**Authors:** Rebecca Rose, Maria Paz Gonzalez-Perez, David Nolan, Krishna Kumar Ganta, Tessa LaFleur, Sissy Cross, Robin Brody, Susanna L. Lamers, Katherine Luzuriaga

**Affiliations:** a BioInfoExperts, LLC, Thibodaux, Louisiana, USA; b UMass Chan Medical School, Worcester, Massachusetts, USA; Emory University School of Medicine

**Keywords:** HIV/AIDS, compartmentalization, evolution, phylogenetics, tropism

## Abstract

HIV-1 sequence population structure among brain and nonbrain cellular compartments is incompletely understood. Here, we compared proviral *pol* and *env* high-quality consensus single-molecule real-time (SMRT) sequences derived from CD3^+^ T cells and CD14^+^ macrophage lineage cells from meningeal or peripheral (spleen, blood) tissues obtained at autopsy from two individuals with viral suppression on antiretroviral therapy (ART). Phylogenetic analyses showed strong evidence of population structure between CD3^+^ and CD14^+^ virus populations. Distinct *env* variable-region characteristics were also found between CD3^+^ and CD14^+^ viruses. Furthermore, shared macrophage-tropic amino acid residues (*env*) and drug resistance mutations (*pol*) between meningeal and peripheral virus populations were consistent with the meninges playing a role in viral gene flow across the blood-brain barrier. Overall, our results point toward potential functional differences among meningeal and peripheral CD3^+^ and CD14^+^ virus populations and a complex evolutionary history driven by distinct selection pressures and/or viral gene flow.

**IMPORTANCE** Different cell types and/or tissues may serve as a reservoir for HIV-1 during ART-induced viral suppression. We compared proviral *pol* and *env* sequences from CD3^+^ T cells and CD14^+^ macrophage lineage cells from brain and nonbrain tissues from two virally suppressed individuals. We found strong evidence of viral population structure among cells/tissues, which may result from distinct selective pressures across cell types and anatomic sites.

## INTRODUCTION

While CD4^+^ T cells clearly play a central role in HIV-1 infection, CD14^+^ macrophages also likely play a role in HIV-1 persistence. Tissue macrophages can harbor productive infection, including in the lung (1–3), gastrointestinal tract (4), brain (5), central nervous system (6), and genitourinary tract (7) of viremic and aviremic individuals. Tissue macrophages are long-lived cells and are resistant to HIV-mediated cytotoxicity (8, 9). Furthermore, while macrophages may overall represent a smaller proportion of infected cells than T cells, particularly in the context of antiretroviral therapy (ART) (10, 11), macrophages (12) and monocytes may harbor a more diverse population than T cells (13, 14) and can contribute to viral rebound when people living with HIV (PLWH) experience therapy interruption (14, 15).

The brain is an early site of HIV-1 infection (16), and ongoing infection of macrophage lineage cells in both the brain and periphery (17) may contribute to the development of neurological disorders (18). The meningeal vascular and lymphatic systems serve as migratory routes of infected cells between the periphery and the brain (reviewed in reference 19). While HIV-1 populations from brain tissue often show compartmentalization with respect to the periphery (20–27), we previously found that meningeal HIV-1 populations harbored viral populations similar to both brain and periphery and found evidence for gene flow between meninges and deeper brain tissue (24, 26).

Compartmentalization of proviral *env* sequences has also been described between blood-derived CD14^+^ monocytes and CD4^+^ T cells from PLWH on (12, 28) or off (13) ART, as well as between blood-derived CD36^+^ monocytes and CD26^+^ T cells from PLWH after therapy interruption (14). On the other hand, virus populations in different T-cell compartments appear to be more mixed, with studies reporting both an absence (29–31) and a presence (32) of population structure among T-cell subsets.

To better understand viral population structure, we compared the proviral *pol* and *env* sequences in T cells (CD3^+^) and macrophage lineage cells (CD14^+^) from meningeal tissues and peripheral blood and/or splenic tissues obtained at autopsy from two individuals with viral suppression on ART.

## RESULTS

### Sequences.

A total of 459,427 reads were obtained from four samples from each participant (Table 1). PCRs of the *pol* region for participant 01-18 were positive only for a single tissue (blood) and were therefore excluded from further analysis. For *env*, each sample resulted in a median of 24,719 reads (range, 1,745 to 119,547). Aligned reads were condensed into a total of 6,942 high-quality consensus sequences (HQCS) (median, 240; range, 7 to 4,257 per sample). These were filtered to a final data set containing a total of 150 HQCS that represented >10 raw reads (HQCS10) (median,14; range, 1 to 68 per sample). For *pol*, each sample resulted in a median of 19,426 reads (range, 12,921 to 70,655). Aligned reads were condensed into a total of 3,306 HQCS (median, 814; range, 9 to 1,669). These were filtered to a final data set containing a total of 76 HQCS10 (median, 13; range, 1 to 50 per sample). All sequences were subtype B.

**TABLE 1:** Sequence numbers^*a*^

Participant	Gene	Tissue	Cell type	DNA used (ng)	No. of CGE	No. of raw reads	No. of filtered reads	No. of HQCS	No. of HQCS10
01-18	*env*	BL	CD3^+^	286	47,667	17,522	15,507	4,527	68
		BL	CD14^+^	682	113,667	19,327	18,794	559	29
		ME	CD3^+^	273	45,500	33,115	32,701	18	1
		ME	CD14^+^	90	15,000	26,258	26,048	39	2
02-16	*env*	BL	CD3^+^	288	48,000	96,307	94,408	31	10
		SP	CD3^+^	912	152,000	23,179	21,690	1,321	17
		SP	CD14^+^	2	333	1,745	1,213	440	20
		ME	CD3^+^	1,044	174,000	119,547	116,941	7	3
	*pol*	BL	CD3^+^	288	48,000	15,476	15,460	1,669	1
		SP	CD3^+^	912	152,000	23,375	21,761	242	23
		SP	CD14^+^	2	333	12,921	12,566	1,386	50
		ME	CD3^+^	1,044	174,000	70,655	69,070	9	2

a BL, blood; ME, meninges; SP, spleen; CGE, cell genome equivalents; HQCS, high quality consensus sequence.

For participant 01-16, the number of cell genome equivalents (CGE) ranged from ~15,000 (meninges CD14) to ~113,000 (blood CD14) (Table 1). For participant 02-16, the number of CGEs ranged from 333 (spleen CD14) to 174,000 (meninges CD3). We also plotted the CGE for each sample and the number of raw/filtered reads, number of HQCS, and number of HQCS10 (see Fig. S1 in the supplemental material). None of the correlations was significant (*P* > 0.25 in all cases). Further, while there was a positive trend between CGE and the number of raw/filtered reads, there was a negative trend between CGE and the number of HQCS/HQCS10.

### Genetic diversity.

**FIG 1:**
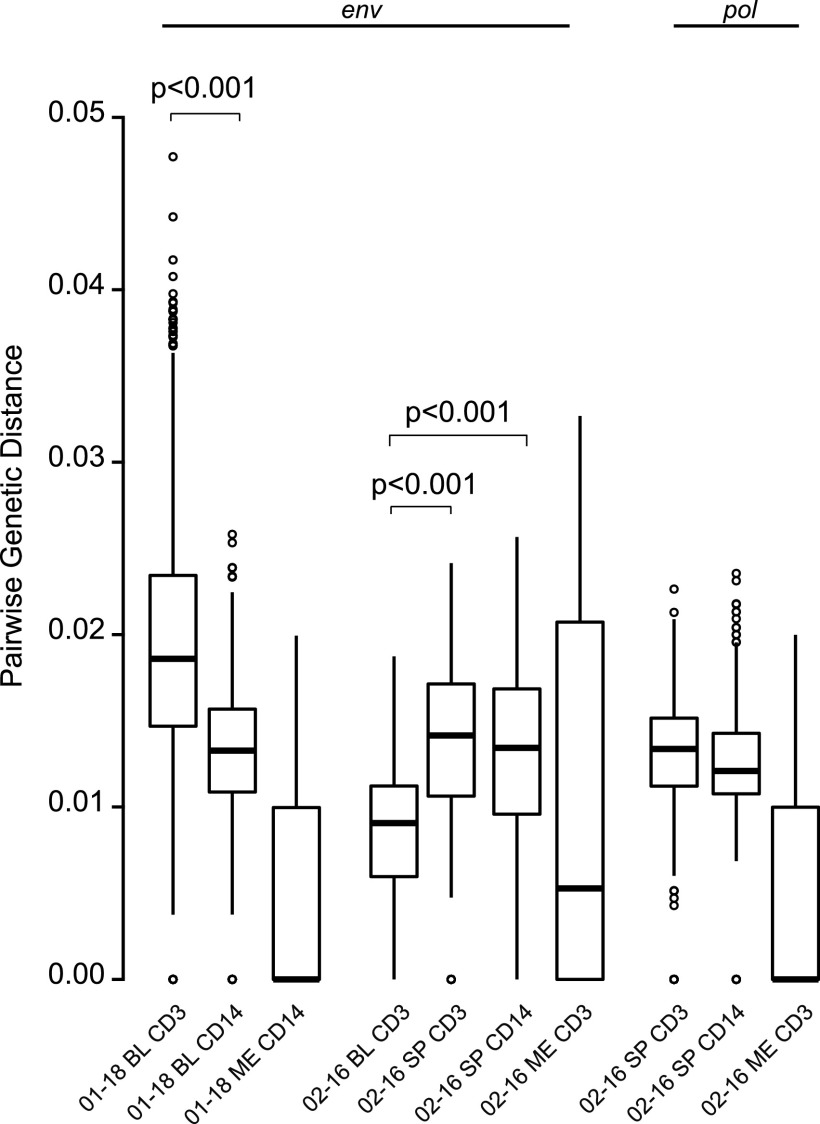
Pairwise genetic diversity for tissue/cells. Boxes indicate the upper and lower interquartile ranges (IQR), with the median represented by a thick horizontal bar. Whiskers extend to 1.5 IQR, and outliers are shown as open circles. Significant comparisons are indicated with a bar and *P* value. BL, blood; ME, meninges; SP, spleen

The median genetic diversity trended lower (although not significantly lower) in participant 01-18 *env*, 02-16 *env*, and 02-16 *pol* (Fig. 1). For 01-18 *env*, diversity was significantly higher in blood CD3^+^ cells than in blood CD14^+^ cells (*P* < 0.001). For 02-16 *env*, diversity was significantly lower in blood CD3^+^ cells than in spleen CD3^+^ (*P* < 0.001) or spleen CD14^+^ (*P* < 0.001) cells. For both 02-16 *env* and 02-16 *pol*, diversity was similar between spleen CD3^+^ and spleen CD14^+^ cells.

### Phylogenies.

**FIG 2:**
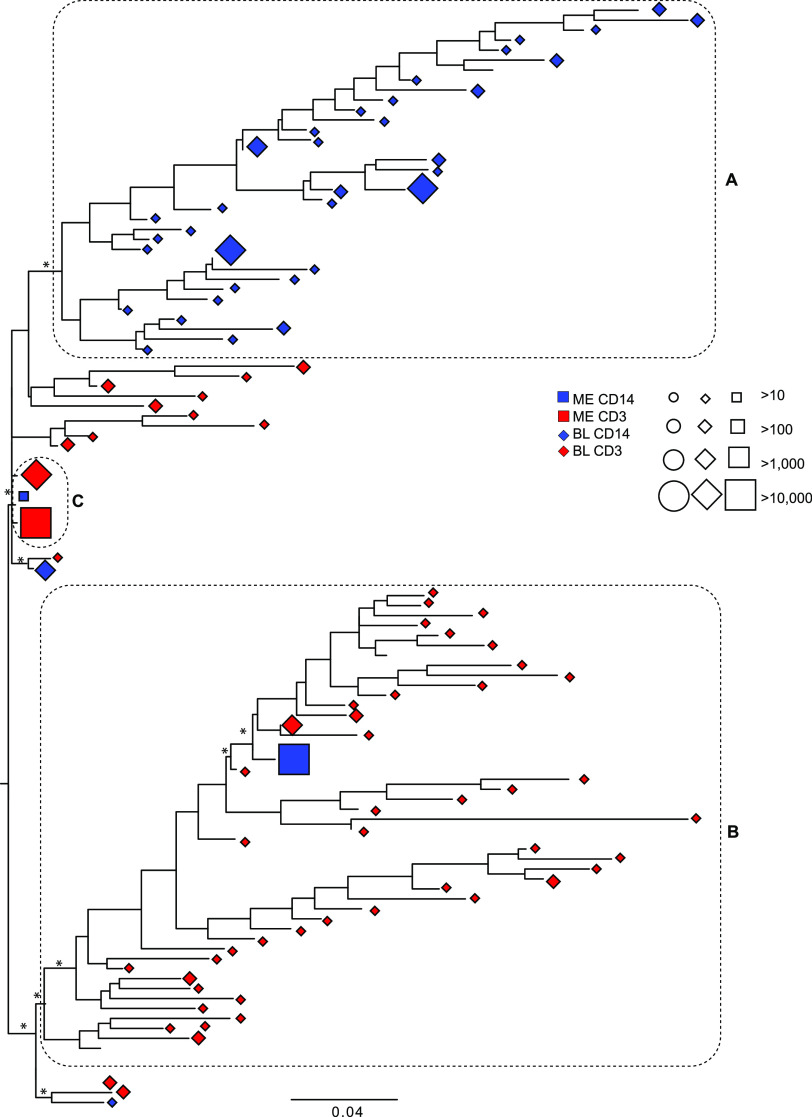
Maximum likelihood phylogeny of *env* HQCS10 variants (excluding variable regions) for participant 01-18. Symbols at the tips represent the variants and the tissue of origin (shape), cellular compartment of origin (color), and number of reads represented (size), according to the legend. Branches are scaled in substitutions per site according to the bar at the bottom. Dotted boxes indicate the clusters of interest discussed in the text. Asterisks indicate branch support of >70%. BL, blood; ME, meninges.

Phylogenies were inferred for *pol* and *env* alignments of the HQCS10 for each participant (excluding the variable regions for *env*). A distinct separation of the blood CD14^+^ and blood CD3^+^ viruses was apparent in the phylogeny for 01-18 *env* (Fig. 2; Fig. S2). A single well-supported cluster (Fig. 2A) contained all but two blood CD14^+^ HQCS10. Another well-supported cluster (Fig. 2B) contained the majority of the blood CD3^+^ HQCS10, which represented ~32% of the raw reads. Interestingly, the meninges CD14^+^ HQCS10 representing >99.9% of the reads clustered with the blood CD3^+^ HQCS10, while the other meninges CD14^+^ HQCS10 was situated close to the single meninges CD3^+^ HQCS10 and a single blood CD3^+^ HQCS10, which represented ~64% of the raw reads (Fig. 2C).

**FIG 3:**
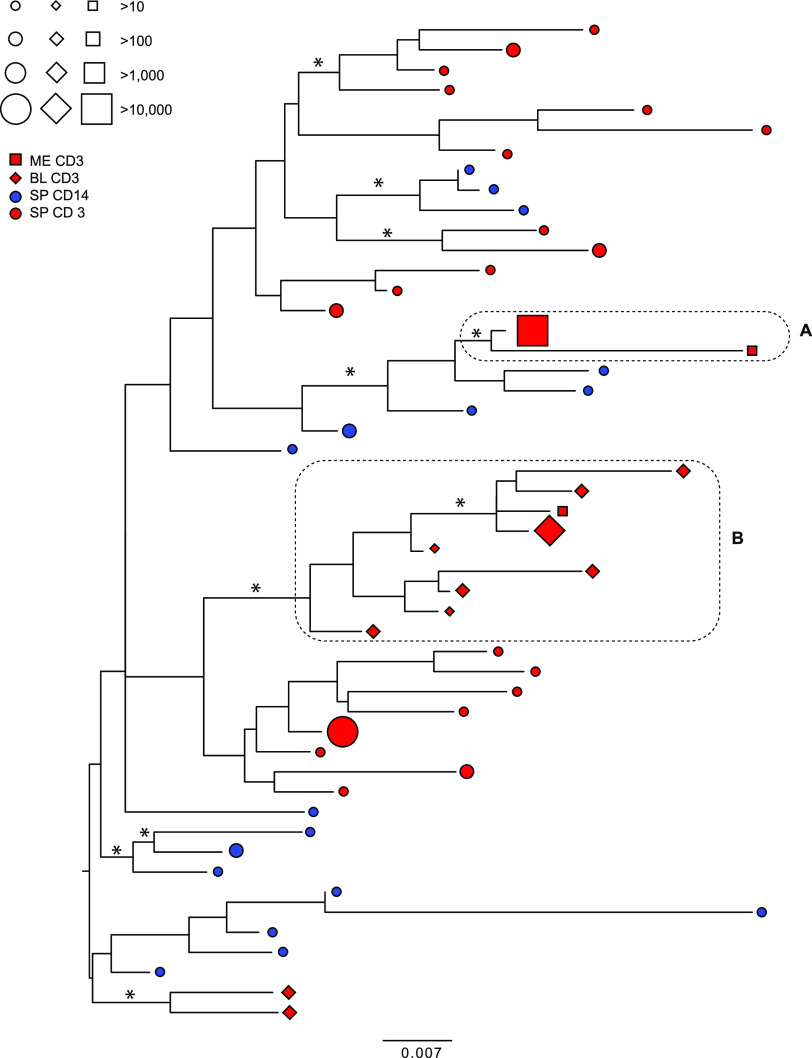
Maximum likelihood phylogeny of *env* HQCS10 variants (excluding variable regions) for participant 02-16. Symbols at the tips represent the variants and the tissue of origin (shape), cellular compartment of origin (color), and number of reads represented (size), according to the legend (ME, meninges; BL, blood; SP, spleen). Branches are scaled in substitutions per site according to the bar at the bottom. Dotted boxes indicate the clusters of interest discussed in the text. Asterisks indicate branch support of >70%.

The *env* phylogeny for participant 02-16 (Fig. 3; Fig. S3) showed a well-supported cluster (Fig. 3A) containing two of the three meninges CD3^+^ HQCS10, the larger of which represented >99% of the reads. This cluster was derived from a larger cluster containing spleen CD14^+^ HQCS10. A single well-supported cluster (Fig. 3B) that represented >99% of the reads contained all but two of the blood CD3^+^ HQCS10, as well as the other minor meninges CD3^+^ HQCS10. The remaining spleen CD3^+^ and CD14^+^ HQCS10 were interspersed throughout the tree, although some degree of separation was evident.

**FIG 4:**
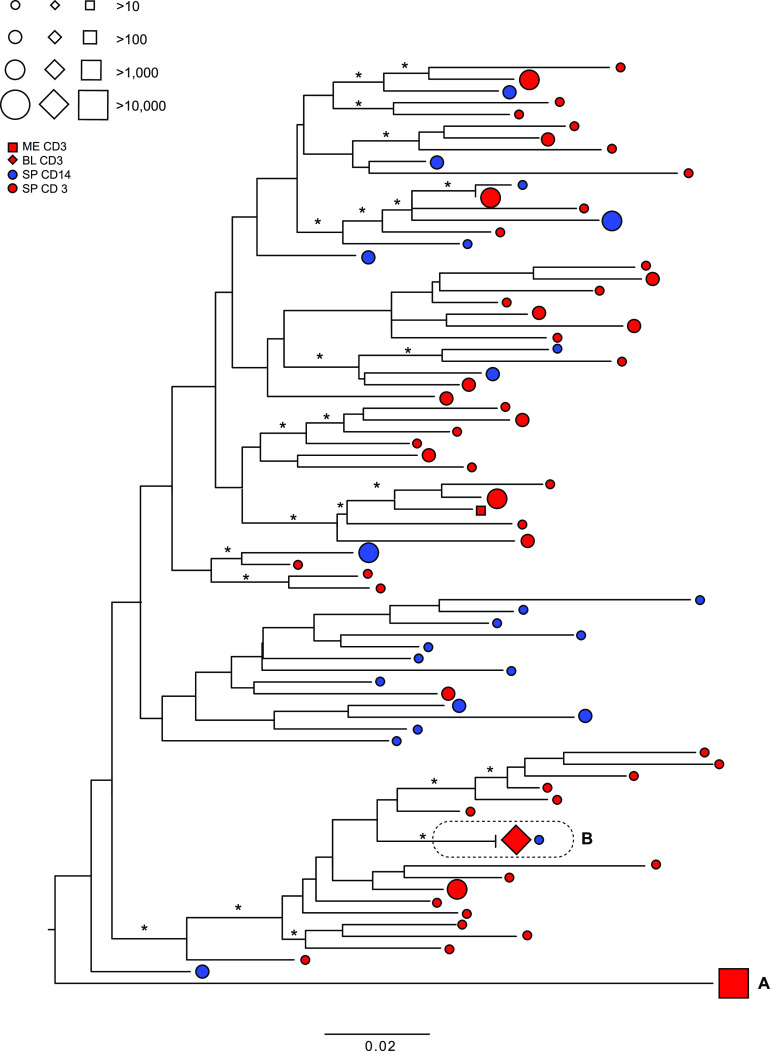
Maximum likelihood phylogeny of *pol* HQCS10 variants for participant 02-16. Symbols at the tips represent the variants and the tissue of origin (shape), cellular compartment of origin (color), and number of reads represented (size), according to the legend (ME, meninges; BL, blood; SP, spleen). Branches are scaled in substitutions per site according to the bar at the bottom. Asterisks indicate branch support of >70%.

In the *pol* phylogeny for participant 02-16 (Fig. 4; Fig. S4), again a single meninges CD3^+^ HQCS10 represented >99% of the reads (Fig. 4A), although here it was placed at the end of a long branch by itself, basal to the rest of the tree. A single blood CD3^+^ HQCS10 represented all of the reads and was identical to a minor spleen CD14^+^ HQCS10 (Fig. 4B). The remaining spleen CD3^+^ and CD14^+^ HQCS10 were again interspersed throughout the tree.

Further investigation of the *pol* HQCS10 showed that virus in the meninges CD3^+^ HQCS10, but not the other tissues, harbored 10 APOBEC3 mutations in the *pol* gene (although these sequences were not identified as hypermutants by the HYPERMUT tool).

### Tests for population structure.

To objectively test for the presence of population structure, we employed a tree-based (Slatkin-Maddison) and non-tree-based (Fst) test for each participant and gene using the maximum likelihood trees and the HQCS10. For both participants, the structure between all CD3^+^ and all CD14^+^ HQCS10 was analyzed. Since only a single tissue from each participant (01-18, blood; 02-16, spleen) had samples from both cellular compartments as well as a sufficient number of branches/HQCS10 for comparison, we also compared the CD3^+^ and CD14^+^ HQCS10 within only blood/spleen tissue. Finally, for 02-16 *env*, we also compared all spleen HQCS10 with all blood HQCS10.

Overall, the tests showed strong support for population structure of CD3^+^ and CD14^+^ HQCS10 (Table 2). For all the comparisons of the *env* HQCS10 in the two participants, the signal for compartmentalization was strongly significant (*P < *0.001). For 02-16 *pol*, a slightly weaker signal for compartmentalization was observed than for *env*, although the *P* value was <0.05 for all tests.

**TABLE 2:** Tests for population structure

Participant	Gene	Tissue or cell comparison	*P* value by indicated Fst metric^*a*^				*P* value by Slatkin-Maddison metric
			HSM	S	HBK	H	
01-18	*env*	CD14^+^ vs CD3^+^	<0.001	<0.001	<0.001	<0.001	<0.001
		CD14^+^ vs CD3^+^ (BL)	<0.001	<0.001	<0.001	<0.001	<0.001
02-16	*env*	CD14^+^ vs CD3^+^	<0.001	<0.001	<0.001	<0.001	<0.001
		CD14^+^ vs CD3^+^ (SP)	<0.001	<0.001	<0.001	<0.001	<0.001
		SP vs BL	<0.001	<0.001	<0.001	<0.001	<0.001
	*pol*	CD3^+^ vs CD14^+^	0.001	0.001	0.001	0.047	0.003
		CD3^+^ vs CD14^+^ (SP)	0.001	0.001	0.001	0.041	0.002

a HSM, Hudson, Slatkin, and Maddison (60); S, Slatkin (61); HBK, Hudson, Boos, and Kaplan (62); H, Hudson nearest neighbor (63).

### Drug resistance mutations.

The HQCS10 *pol* sequences for participant 02-16 were analyzed using the HIVdb tool (Table 3). For the nucleoside reverse transcriptase inhibitors (NRTI), meninges CD3^+^ HQCS10 were predicted to have high-level resistance to lamivudine (3TC; part of this individual’s ART regimen) and emtricitabine (FTC) and low-level resistance to abacavir (ABC; part of this individual’s ART regimen). Blood CD3^+^ HQCS10 were predicted to have high-level resistance to zidovudine (AZT), intermediate resistance to ABC and tenofovir (TDF), and potential low-level resistance to FTC and 3TC. None of the spleen CD3^+^ or CD14^+^ HQCS10 were predicted to have NRTI resistance.

**TABLE 3:** Resistance profiles for participant 02-16

Drug class^*a*^	Drug abbrev^*b*^	Resistance in indicated cells			
		ME CD3^+^	BL CD3^+^	SP CD3^+^	SP CD14^+^
NRTI	ABC	Low level	Intermediate	Susceptible	Susceptible
	AZT	Susceptible	High level	Susceptible	Susceptible
	FTC	High level	Low level^*c*^	Susceptible	Susceptible
	3TC	High level	Low level^*c*^	Susceptible	Susceptible
	TDF	Susceptible	Intermediate	Susceptible	Susceptible
NNRTI	DOR	Susceptible	Susceptible	Susceptible	Susceptible
	EFV	Susceptible	High level	Susceptible	Susceptible
	ETR	Susceptible	Susceptible	Susceptible	Susceptible
	NVP	Susceptible	High level	Low level	Susceptible
	RPV	Susceptible	Susceptible	Susceptible	Susceptible
ISTI	BIC	Susceptible	Susceptible	Susceptible	Susceptible
	CAB	Susceptible	Susceptible	Susceptible	Susceptible
	DTG	Susceptible	Susceptible	Susceptible	Susceptible
	EVG	Susceptible	Susceptible	Susceptible	Susceptible
	RAL	Susceptible	Susceptible	Susceptible	Susceptible

a NNRT, nucleoside reverse transcriptase inhibitors; NNRTI, nonnucleoside reverse transcriptase inhibitors; ISTI, integrase strand transfer inhibitors.

b ABC, abacavir; BIC, bictegravir; CAB, cabotegravir; DTG, dolutegravir; DOR, doravirine; EFV, efavirenz; EVG, elvitegravir; FTC, emtricitabine; ETR, etravirine; 3TC, lamivudine; NVP, nevirapine; RAL, raltegravir; RPV, rilpivirine; TDF, tenofovir; AZT, zidovudine. Drugs in bold were part of the ART regimen for this participant.

c Potential low level.

For the nonnucleoside reverse transcriptase inhibitors (NNRTI), none of the meninges CD3^+^ HQCS10 were predicted to have resistance. Blood CD3^+^ HQCS10 were predicted to have high-level resistance to efavirenz (EFV) and nevirapine (NVP). Spleen CD3^+^ HQCS10 were also predicted to have low-level resistance to NVP. None of the spleen CD14^+^ HQCS10 were predicted to have NNRTI resistance. No HQCS10 from any tissue was predicted to have resistance to integrase strand transfer inhibitors (ISTI), including dolutegravir (DTG), which was also part of this individual’s ART.

Specific drug-resistant mutations were also identified for each sample (Table 4). Meninges CD3^+^ HQCS10 had the M184V NRTI mutation, while the blood CD3^+^ HQCS10 had the D67N, K70R, T215F, and K219E NRTI mutations. Blood CD3^+^ HQCS10 also had the K103N NNRTI mutation, and both blood and spleen CD3^+^ HQCS10 had the N348I NNRTI mutation. In addition, meninges CD3^+^ HQCS10 had one APOBEC mutation in the reverse transcriptase (RT) gene (D549N) and nine in the integrase (IN) gene (E13K, D25N, G47E, W61, E69K, G82E, E85K, E157K, and R166K).

**TABLE 4:** Significant mutations for 02-16 *pol*

Mutation type	Mutation(s) in indicated sequence			
	ME CD3^+^	BL CD3^+^	SP CD3^+^	SP CD14^+^
NRTI	M184V	D67N, K70R, T215F, K219E	None	None
NNRTI	None	K103N, N348I	N348I	None
IN (major)	None	None	None	None
IN (minor)	None	None	None	None
RT APOBEC	D549N			
IN APOBEC	E13K, D25N, G47E, W61, E69K, G82E, E85K, E157K, R166K			

### *env* variable-region analysis.

Four characteristics (predicted tropism, charge, number of glycosylation sites, and length) were measured for each read in the five variable regions of *env*. For all tissues/cells, the vast majority of reads (>99%) were predicted to use the CCR5 coreceptor (Table 5). There was a slightly higher percentage of reads (0.8%) predicted to use the CXCR4 coreceptor for 01-18 blood CD3^+^.

**TABLE 5:** Predicted tropism for the number (percent) of reads for each sample^*a*^

Participant	Tissue	Cell type	No. (%) of reads	
			R5	X4
01-18	BL	CD3^+^	14,206 (99.2)	115 (0.8)
		CD14^+^	18,926 (>99.99)	2 (<0.01)
	ME	CD3^+^	32,476 (>99.99)	1 (<0.01)
		CD14^+^	25,989 (100)	0 (0)
02-16	BL	CD3^+^	47,377^*b*^ (>99.99)	1 (<0.01)
	SP	CD3^+^	23,040 (>99.99)	2 (<0.01)
		CD14^+^	1,726 (100)	0 (0)
	ME	CD3^+^	35,225^*b*^ (>99.99)	3 (<0.01)

a R5, Predicted to use the CCR5 co-receptor; X4, Predicted to use the CXCR4 co-receptor.

b Subsampled at 50%.

**FIG 5:**
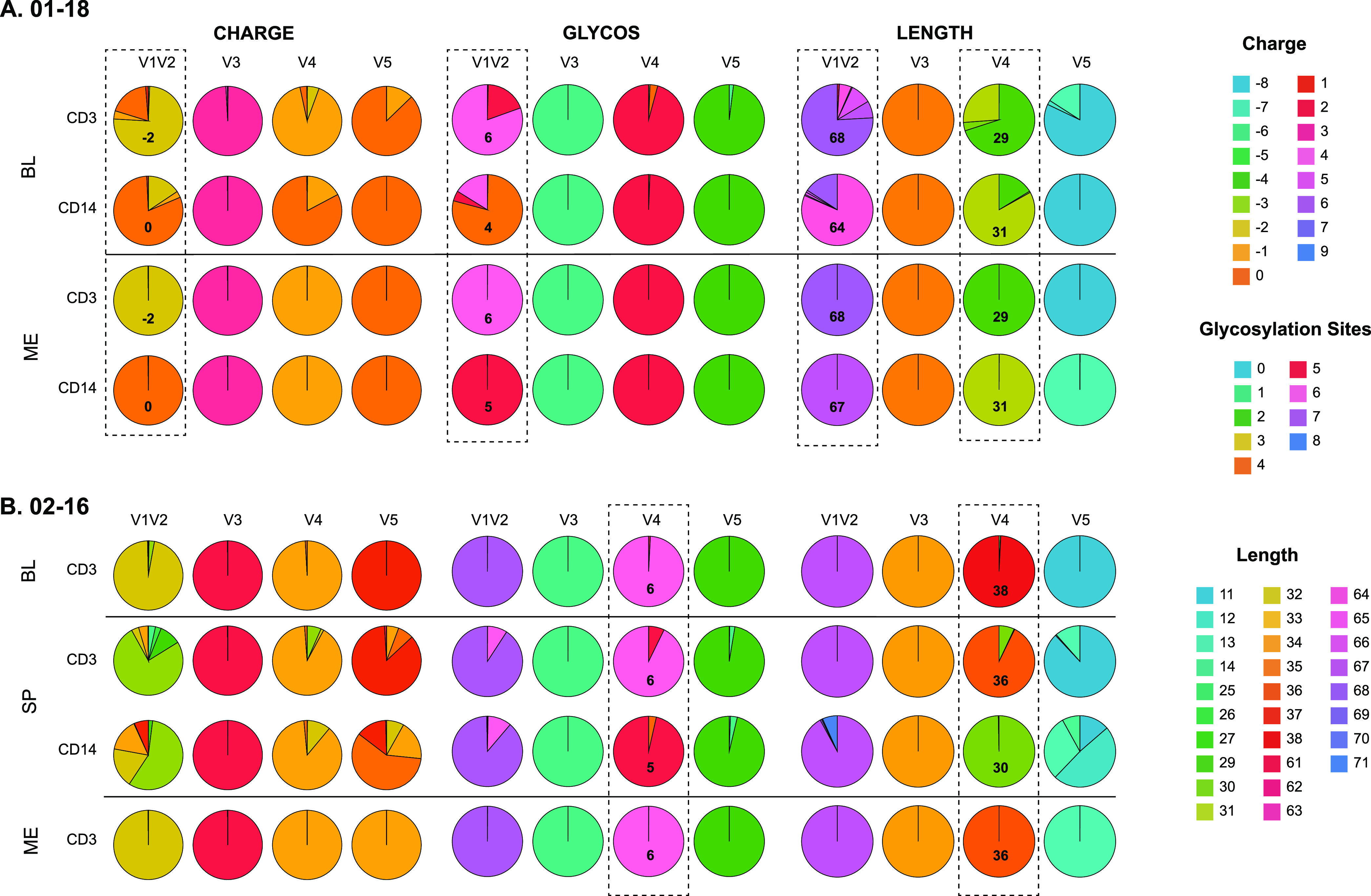
Variable region characteristics (charge, number of glycosylation sites, and length) for participants 01-18 (A) and 02-16 (B) by tissue/cell compartment. The relative sizes of the pie slices represent the number of reads with each value. Values are indicated by color according to the legend on the right and in some cases noted in the pie slice representing the majority of reads. Dotted boxes indicate the patterns of interest discussed in the text. ME, meninges; BL, blood; SP, spleen.

Charge, length, and number of glycosylation sites were homogeneous in the meninges CD3^+^ populations for both participants and in the meninges CD14^+^ population for participant 01-18 (Fig. 5). On the other hand, the blood CD3^+^ and CD14^+^ populations (for participant 01-18) and the spleen CD3^+^ and CD14^+^ populations (for participant 02-16) showed more diversity, particularly in V1V2 and V4. Interestingly, the charge and glycosylation site profiles had striking similarities between the blood and meninges CD3^+^ samples and the blood and meninges CD14^+^ samples for participant 01-18 and between the blood, meninges, and spleen CD3^+^ samples and the spleen CD14^+^ samples for participant 02-16. For example, in participant 01-18, the majority of V1V2 reads from CD3^+^ cells in blood (~80%) and meninges (>99%) had a −2 charge, 6 glycosylation sites, and a length of 68 amino acids (aa), whereas the majority of V1V2 reads from CD14^+^ cells in blood (~75%) and meninges (>99%) had a 0 charge, 4 or 5 glycosylation sites, and a length of 64 to 67 aa (Fig. 5A). Similarly, the majority of V4 reads from CD3^+^ cells in blood (~70%) and meninges (>99%) had a length of 29 aa, while a majority of V4 reads from CD14^+^ cells in blood (~83%) and meninges (>99%) had a length of 31 aa. No clear differences were observed in the other variable regions. This pattern in the variable regions for CD3^+^ mirrors the phylogeny, where the most frequent HQCS10 for blood and meninges, representing ~64% and >99% of the reads, respectively, cluster closely together. On the other hand, the meninges and blood CD14^+^ HQCS10 are situated in different parts of the tree.

A similar pattern was observed for V4 for participant 02-16: the majority of V4 reads from CD3^+^ cells in blood (>99%), meninges (>99%), and spleen (~93%) had 6 glycosylation sites and a length of 36 to 38 aa, whereas the majority of V4 reads from CD14^+^ cells in spleen (>97%) had 5 glycosylation sites and a length of 30 to 67 aa (Fig. 5B). This pattern in the variable regions for 02-16 is only somewhat consistent with the pattern observed in the phylogeny: while the blood CD3^+^ HQCS10 grouped most closely to spleen CD3^+^ HQCS10, the meninges CD14^+^ HQCS10 were mostly closely related to spleen CD14^+^ HQCS10 and grouped separately from the blood/spleen CD3^+^ HQCS10.

**FIG 6:**
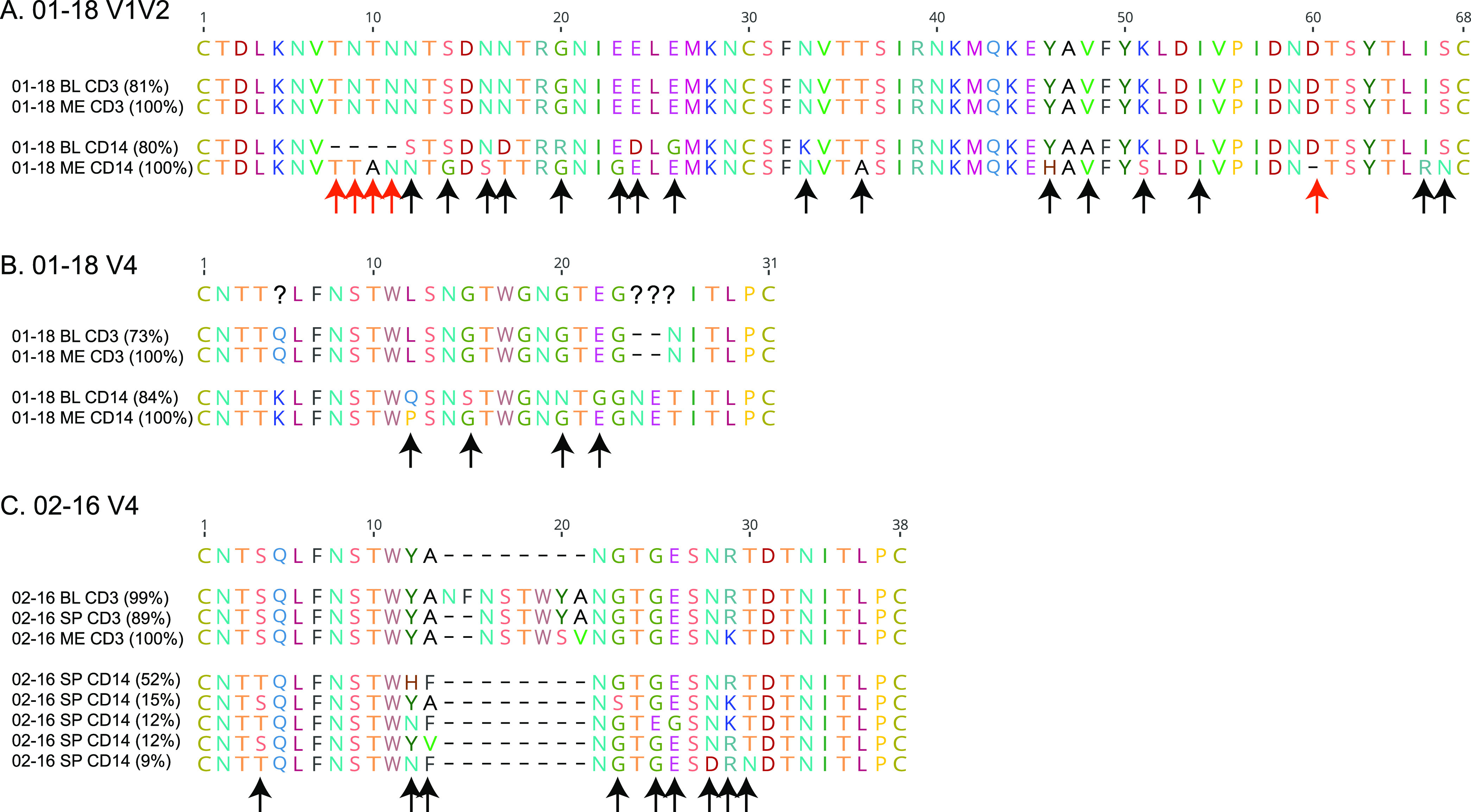
Majority variants for 01-18 V1V2 (A), 01-18 V4 (B), and 02-16 V4 (C) regions. The variant representing the most reads (at 100% identity) is shown for each tissue/cell (ME, meninges; BL, blood; SP, spleen). The percentage of reads represented by each variant is shown in parentheses. The position of the residues relative to the longest sequence is shown for each alignment. Arrows indicate positions where the CD14^+^ sequences have a substitution (black) or an insertion/deletion (red) relative to each other.

To investigate this further, we clustered the V1V2 and V4 translated reads at 100% identity and retained the variants that represented >100 reads for each tissue/cell (Fig. S5). We then compared the majority variants (i.e., the variants that represented >50% reads) for each tissue/cell (Fig. 6). For participant 01-18, the V1V2 majority variants from blood and meninges CD3^+^ cells were identical (Fig. 6A), which is consistent with the close proximity of their HQCS10 on the phylogenetic tree. On the other hand, the V1V2 majority variants from blood and meninges CD14^+^ cells showed 16 amino acid differences, plus five insertion/deletions, relative to each other. Similarly, the V4 majority variants from blood and meninges CD3^+^ cells were identical (Fig. 6B). While the V4 majority variants from blood and meninges CD14^+^ cells shared some characteristics (e.g., the K at position 5, the insertion of NE at positions 24 and 25, and the N at position 26 relative to the CD3^+^ majority variants), they also showed four amino acid differences between them. Interestingly, at three of those four sites, the amino acid in the meninges CD14^+^ majority variant was the same as the amino acid in the meninges CD3^+^ majority variant.

For participant 02-16, a similar pattern was seen for the V4 majority variants (Fig. 6C). The majority variants from blood, spleen, and meninges CD3^+^ cells were similar (blood majority variants had an NF insertion at positions 14 and 15, and meninges majority variants had one amino acid difference relative to the other two). On the other hand, the spleen CD14^+^ majority variant had two amino acid differences plus a 6- to 8-amino-acid deletion relative to the CD3^+^ sequences. However, since the spleen CD14^+^ majority variant represented only 51% of the reads, we included the other variants that represented >100 reads as well. Further inspection showed that all of the spleen CD14^+^ variants shared the deletion, although they showed up to five amino acid differences among them.

Finally, we looked at the amino acids in positions previously reported as being associated with macrophage or T cell tropism (Table 6). For participant 01-18, a glycine was found at position 153 (V1) in all meninges CD14^+^ sequences, while an asparagine was found at position 362 (C3) in all blood CD14^+^ and meninges CD3^+^ sequences and in 75% of blood CD3^+^ sequences but in none of the meninges CD14^+^ sequences. In participant 02-16, two macrophage-tropic residues were found in all sequences from all cells/tissues: an asparagine at position 188 in V2 and an aspartic acid at position 279 in C2. In addition, an asparagine was found at position 283 in C2 for all meninges CD3^+^ sequences and 75% of spleen CD14^+^ sequences.

**TABLE 6:** Amino acid positions associated with tropism^*a*^

Tissue/cell origin	Amino acid in indicated region at indicated position											
	V1	V2		C2		V3		C3				V4
	153	178	188	279	283	308	317	362	363	364	373	386
T cell	E	K	T	N	T/I/V	H/T	F	T	Q	P	R	N
Macrophage	G	E	N	D	N	N/P	L	N	P	S	K	D
01-18 BL CD3	E	K	T	N	V	R	F	**N**/T^*b*^	H	S	M/L	N
01-18 BL CD14	E	K	T	N	V	R	F	**N**	H	S	M	N
01-18 ME CD3	E	K	T	N	V	R	F	**N**	H	S	M	N
01-18 ME CD14	**G**	S	T	N	V	R	F	T	H	S	M	N
02-16 BL CD3	E	K	**N**	**D**	V	H	F	Q	E	S	M	N
02-16 SP CD3	E	K	**N**	**D**	V	H	F	Q/E	E/K	S	M	N
02-16 ME CD3	E	K	**N**	**D**	**N**	H	F	Q/E	E/K	S	M	N
02-16 SP CD14	E	K	**N**	**D**	V/**N**^*c*^	H	F	K/E	E/K	S	M	N

a Adapted from reference 33. Boldfaces indicate the presence of an amino acid substitution previously associated with macrophage tropism.

b N, 75%; T, 25%.

c V, 75%; N, 25%.

## DISCUSSION

In this study, we used an ultradeep sequencing approach to characterize the genetic diversity of the *env* and *pol* genes from HIV-1 provirus isolated from CD3^+^ and CD14^+^ cells derived from meningeal and peripheral tissues of two individuals with viral suppression on ART, neither of whom showed clinical neuropathology at death. We found evidence of population structure between CD3^+^ and CD14^+^ virus populations by using multiple methods, as well as evidence of possible functional differences between viral populations.

All phylogenetic trees showed a single HQCS10 representing >99% of reads in all meningeal tissues available (CD3^+^ and CD14^+^ in participant 01-18; CD3^+^ in participant 02-16). Somewhat surprisingly, in the *env* phylogenies, two of these meninges HQCS10 clustered most closely with HQCS10 from the noncorresponding cell type in the periphery (01-18, meninges CD14^+^ with blood CD3^+^; 02-16, meninges CD3^+^ with spleen CD14^+^), while only the meninges CD3^+^ HQCS10 in participant 01-18 clustered with the corresponding blood CD3^+^ HQCS10. In the 02-16 *pol* phylogeny, the majority meninges HQCS10 was situated on a long branch basal to the rest of the tree. This low viral diversity in the meninges compared to that in the blood and spleen is intriguing; however, the lack of input cell copy number prevents a direct comparison. We therefore calculated the estimated number of CGE for each sample, which approximates the number of input cells. For participant 01-16, the number of CGE in meninges CD14 samples was the lowest (~15,000) among all samples, although the number of CGE in meninges CD3 samples (~45,000) was similar to that in blood CD3 samples (~48,000). For participant 02-16, the number of CGE in meninges CD3 samples (~174,000) was the highest of all the samples. These results may suggest that the meninges is a distinct microenvironment with respect to the periphery, although additional studies are necessary to confirm this hypothesis.

Interestingly, in both participants, HQCS10 from peripheral CD3^+^ and CD14^+^ cells (01-18, blood; 02-16, spleen) showed evidence of intracellular clustering. Multiple statistical tests confirmed significant population structure between CD3^+^ and CD14^+^ populations. Furthermore, we found distinct amino acid patterns between the two cell types in the V1V2 and V4 regions. On the surface, these shared characteristics appear to be inconsistent with the phylogenies for the two participants. For example, in participant 01-18, the phylogeny showed that the meninges CD14^+^ majority variant was more closely related to the blood CD3^+^ virus than the blood CD14^+^ virus, while in participant 02-16, a population of spleen CD14^+^ virus was most closely related to the meninges CD3^+^ majority variant. Closer examination of the amino acid sequences from 01-18 showed that despite sharing similar characteristics, the V1V2 and V4 sequences from the two CD14^+^ populations were clearly distinct: in V4, the meninges CD14^+^ majority variant was in fact the same distance from the blood CD14^+^ majority variant as it was from the meninges CD3^+^ majority variant. For 02-16, inspection of the V4 amino acid sequence showed that the spleen CD14^+^ population actually contained multiple variants, which differed by up to five amino acids while maintaining the same length (8 amino acids shorter than the spleen CD3^+^ major variant). This may suggest that the deletion occurred independently in multiple populations.

Interestingly, several amino acid residues previously found to be associated with macrophage tropism and/or CD4 binding (33) were detected. In participant 01-18, G153 (V1) was found in all meningeal CD14^+^ sequences, but in none of the other cells/tissues. In contrast, N362 (C3) was found in all blood CD14^+^ and meninges CD3^+^ sequences and in 75% of the blood CD3^+^ sequences but none of the meninges CD14^+^ sequences. In participant 02-16, macrophage-tropic residues were found in three positions, two of which were shared by all sequences (N188, V2; D279, C2), and the other was found in both meninges CD3^+^ and spleen CD14^+^ sequences (N283, C2). Positions 279, 283, and 362 are located within/immediately adjacent to the CD4-binding region (34). The presence of an asparagine at position 283 has been associated with brain-derived envelopes, increased *env*-CD4 affinity, and enhanced macrophage tropism (35). The aspartic acid at position 279 and the asparagine at position 362 have been shown to reduce dependency on CD4 and increase fusogenicity, a prominent phenotype of R5 viruses (34). Although analysis of the V3 loop predicted that <1% of sequences used the CXCR4 coreceptor, it is possible that determinants outside of the V3 loop have conferred some degree of macrophage tropism. Furthermore, it is interesting that the residues associated with macrophage tropism were not confined to just the CD14^+^ viruses (with the exception of G153 in 01-18 meninges CD14^+^ virus). This is consistent with studies showing that residues act synergistically, so that the presence of a single residue may not confer a significant functional difference (34, 35). Furthermore, the shared N283 residue for the meninges CD3^+^ and spleen CD14^+^ viruses might suggest that viruses in meningeal T-cell populations have functional characteristics that confer an advantage in a CD4-reduced environment. Furthermore, there was a strikingly different predicted resistance profile for viruses in the different tissues: meninges and blood, but not spleen, showed resistance mutations against the two NRTIs that were part of the participant’s ART regimen. High-level resistance was predicted for an additional two NRTIs (blood, AZT; meninges, FTC) and two NNRTIs (blood, EFV and NVP) that were not part of the individual’s ART regimen at the time of death and may have resulted from previous exposure to these drugs. Unfortunately, a complete history of this participant’s ART regimen was unavailable, so we are unable to confirm this hypothesis.

The presence of 10 putative APOBEC3 mutations in the meninges CD3 HQCS10 from participant 02-16 was interesting. APOBEC3 proteins catalyze the deamination of cytosine to uracil during reverse transcription, resulting in G-to-A mutations (36, 37). Although high levels of APOBEC3 mutations may impede viral replication and result in defective viruses, these viruses could still elicit immune responses and/or produce proteins (38, 39), while sublethal levels of APOBEC3 mutations may actually increase viral fitness by facilitating immune escape (40). Further investigation of these mutations in the meninges CD3^+^ HQCS10 is required to assess their potential functional relevance.

One potential explanation for the observed results is experiment-induced mutations and/or recombination. The error rate of the unprocessed long reads from the single-molecule real-time (SMRT) platform is higher than that of short reads produced from other sequencing platforms. However, the circularization of the amplicon during sequencing, known as circularized consensus sequencing (CCS), allows numerous polymerase passes of the same amplicon, from which a consensus sequence is generated with much lower error rates (41, 42). Previous studies have calculated an error rate of 0.02% using seven full CCS passes (43). Here, we used 15 complete amplicon passes as our minimum for inclusion, out of an abundance of caution to limit the influence of sequence error. Furthermore, the majority of sequencing errors using this technology are insertions and deletions concentrated in homopolymeric regions (44). Our manual removal of sites with >95% gaps further reduced the influence of sequence error in our alignments and subsequent analyses. Finally, clustering the reads and using the HQCS10 variants only should drastically limit the impact of errors on the analysis. To limit the potential impact of experiment-induced recombination, we used a high-fidelity polymerase and increased PCR extension times, although we recognize that these measures may not entirely eliminate recombinant reads. However, Laird Smith and coauthors reported a recombination rate of <1% in their SMRT-derived data set, suggesting that recombination may not impact the results to a substantial degree (44).

The inherent difficulty in obtaining human brain tissue is reflected in the small sample size of this study and the lack of a complete clinical history for the participants, which may limit the ability to translate these findings to other contexts. We also note that another limitation of this study is the reliance on a single sequencing method that uses bulk PCR as input. During bulk PCR, minor variants may be preferentially amplified and become overrepresented in the sequences, causing other major/minor variants to be underrepresented (or entirely absent). Therefore, the frequencies of the viral variants we observed are not necessarily a true reflection of the underlying frequency in the population, nor do they necessarily represent the entirety of the viral diversity for each tissue/cell, and we caution against overinterpretation of the results. On the other hand, in the absence of a systematic bias (e.g., primer bias) against specific variants, it seems unlikely that such an imbalanced amplification would have occurred in the majority of samples, thus giving a false signal of population structure. Nevertheless, the case for SMRT sequencing (and all newer high-throughput sequencing methods) would strongly benefit from additional studies that carefully and systematically compare them with more traditional methods such as single genome sequencing.

There is ongoing debate about the degree to which monocytes are productively infected (11, 45), which is due in part to questions about the purity of sorted cell populations from which virus is derived (11) and the specific subset of monocytes under investigation (46). Unfortunately, limited cell numbers from autopsy tissues precluded flow cytometric studies to confirm the purity of the cell populations in the present study. Our observation that macrophage-tropic mutations were incompletely associated with meninges-derived sequences could have resulted from mixed cell populations. On the other hand, the methodology we used here (positive cell selection using magnetic beads) has been assessed in previous studies that found >98% cell purity for both CD3^+^ (47) and CD14^+^ (48, 49) populations, providing indirect support for the robustness of the method.

In summary, the patterns that we observed in this study are intriguing, although the limitations of this study discussed above prevent firm conclusions from being drawn. Additional studies should provide further insight into HIV-1 sequences and function in cell-based reservoirs.

## MATERIALS AND METHODS

### Study participants.

Postmortem blood and tissues from two individuals residing in the United States were provided by the National Neuro-AIDS Tissue Consortium (NNTC) and the National Disease Research Interchange (NDRI) without patient-identifying information. Clinical and laboratory findings on the individuals studied are summarized in Table 7, including CD4 count, plasma, and cerebrospinal fluid (CSF) viral load and ART regimen at the time of death. Both individuals were on combination antiretroviral therapy; plasma viral load was undetectable (<20 RNA copies/mL) by clinical assay in one individual (02-16), while plasma viral load was very low (580 RNA copies/mL) in the second individual (01-18) studied. Neither individual had clinical neuropathology at death. The UMass Chan School IRB considered that this research was not human subject research as defined by DHHS and FDA regulations (IRB docket no. H00014098).

**TABLE 7:** Clinical and laboratory data on study participants

PID^*a*^	Age (yr)	Gender	Race	Cause of death (yr)	Plasma RNA (copies/mL)	Absolute CD4 (cells/mm^3^)	Duration of infection (yr)	ART^*c*^ at time of death	Past ART
01-18	63	Male	White	NA^*b*^ (2018)	580	853	34	DRV/c, FTC, TDF^*d*^	AZT, ddl, ddc, 3TC, FTC, TDF, RTV, NFV, EFV
02-16	64	Male	Black	Cardiac arrest (2016)	<20	NA	22	ABC, DTG, 3TC	NA

a PID, participant identifier.

b NA, not available.

c ABC, abacavir; DTG, dolutegravir; 3TC, lamivudine; FTC, emtricitabine; TDF, tenofovir; AZT, zidovudine; ddl, didanosine; ddc, zalcitabine; EFV, efavirenz; RTV, ritonavir; NFV, nelfinavir; DRV/c, darunavir-cobicistat.

d Last dose at 52 h premortem.

### Blood and tissue specimens.

A specimen from the meninges and at least one specimen from peripheral blood or spleen were collected from each individual at autopsy. Whole blood was first centrifuged to remove the plasma, after which peripheral blood mononuclear cells (PBMC) were isolated by Ficoll separation (Sigma Millipore). Splenic and meningeal tissues were processed into single-cell suspensions, prior to cell sorting. CD3^+^ T cells and CD14^+^ macrophage lineage cells were isolated by sequential positive selection (CD3^+^ then CD14^+^) from PBMC and splenic or meningeal single-cell suspensions using magnetic activated cell sorting (MACS), per the manufacturer’s protocol (Miltenyi). The estimated number of cells (cell genome equivalents [CGE]) was calculated based on the amount of DNA used for amplifications using the ratio of 6 pg DNA/cell (50, 51).

### Nucleic acid isolation and PCR HIV-1 *env* amplification.

Genomic DNA was isolated from each sample using a QIAamp DNA minikit (Qiagen) as described in the manufacturer’s protocol. DNA was eluted in nuclease-free, PCR-grade water and stored at −80°C until analysis. Genomic DNA was quantified using the Qubit 3.0 fluorimeter (Thermo Fisher Scientific) (Table 1). A bulk nested-PCR approach was used to amplify an ~3-kb full-length *env* product as previously described (22). For *pol* amplification, a bulk nested-PCR approach was used to amplify a 2.5-kb product from tissue DNA. Outer primers RT18.5 (bp 2396 to 2430 relative to HXB2; 5′-GGGAATTGGAGGTTTTATCAAAGTAAGACAGTATG-3′) and intR1 (bp 5051 to 5080; 5′-CTACCTGCCACACAATCATCACCTGCCATC-3′) (52) and inner primers RT19.5 (bp 2484 to 2512; 5′-GGACCTACACCTGTCAACATAATTGGAAG-3′) and intR2 (bp 4956 to 4984; 5′-TGTATTACTACTGCCCCTTCACCTTTCCA-3′) (52) at a 0.2 μM final concentration were used. The PCR protocol was as follows: 95°C for 1 min and then 30 cycles of 95°C for 30 s, 68°C for 3 min, and 70°C for 10 min. A second PCR round of 40 cycles was then undertaken, using the same conditions. The PrimeSTAR GXL DNA polymerase (TaKaRa) was used as described in the manufacturer’s protocol for 50-μL reaction mixtures. To reduce PCR-mediated recombination, we increased our extension times to 3 min to allow more time for completion of each amplicon before denaturation. PCR products were imaged on a 2% agarose gel with GelGreen nucleic acid gel stain (Biotium) using a blue light transilluminator and purified by use of a Zymoclean gel DNA recovery kit (Zymo). PCR products were eluted in 8 μL of nuclease-free, PCR-grade water and sent to the Interdisciplinary Center for Biotechnology Research core sequencing laboratory at the University of Florida for library preparation and single-molecule real-time (SMRT) sequencing using the PacBio Sequel instrument. The LR v3 SMRT cell was utilized with 20-h collection times for each run, with 4 to 8 libraries multiplexed in a single SMRT cell.

### SMRT sequence processing.

SMRT sequence data were processed for each sample/gene separately using methods adapted from Laird Smith and coauthors (44) and used by our group previously (20, 22). SMRT cell raw reads for each tissue were selected to include only circular consensus reads of >2,000 bp with at least 15 complete amplicon passes and quality filtered at 99.99% read quality using the tool bamtools filter. Reads were then assembled to a single-endpoint-dilution, participant-specific reference sequence for either *env* or *pol* by using the Pacific Biosciences tool pbmm2 (v.1.7.0).

The region containing the majority of the reads was extracted and gap stripped to remove spurious inserts, with columns with ≥95% coverage retained using Geneious Prime software (https://www.geneious.com/). The alignment was manually optimized using AliView (v.1.17.1) (53) (e.g., sequences with >100 bp missing compared to the participant-specific reference sequence; nonhomologous variable regions [V1 to V5] were removed). Reads were then clustered at 99% genetic identity using USEARCH v10.0.24 (54), and the centroid sequence of each cluster was assigned as the HQCS to correct for sequencing errors that may have resulted due to the SMRT sequencing technology (44). To further reduce the impact of potential sequencing error, only HQCS that represented >10 raw reads (HQCS10) were retained for phylogenetic/distance analyses (Table 1). Correlation analysis between the number of raw reads, filtered reads, HQCS, HQCS10, and starting amount of DNA was performed in R (55).

### Sequence analysis.

Sequences were subtyped using the Rega HIV-1 subtyping tool (http://dbpartners.stanford.edu:8080/RegaSubtyping/stanford-hiv/typingtool/). Phylogenetic trees were inferred for *env* and *pol* by using participant-specific HQCS10 variants. Trees were generated using the Hasegawa-Kishino-Yano nucleotide model of substitution (56) with gamma-distributed, among-site rate variation. One thousand ultrafast bootstraps were generated to assess branch support using IQTREE v2 (57). Trees were midpoint rooted for ease of visualization, although since the sequences from each participant were obtained from the same time point, no inference of time and/or directionality should be inferred.

Hypermutation was analyzed using the HYPERMUT tool (https://www.hiv.lanl.gov/content/sequence/HYPERMUT/hypermut.html) using the HXB2 subtype B sequences as the reference. Resistance mutations were identified by analyzing *pol* sequences using the HIVdb tool (https://hivdb.stanford.edu/hivdb). Pairwise diversity among HQCS10 was calculated using the *tn93* tool (https://github.com/veg/tn93). Significance for genetic distance among groups was assessed using the pairwise Wilcoxon rank sum test in R (package sjstats). Population structure among tissues/cellular compartments was assessed with HYPHY (58) using both a distance-based method (Fst) and a tree-based method (Slatkin-Maddison [59]). Four Fst metrics were calculated (Hudson et al. [60], Slatkin [61], Hudson et al. [62], and Hudson [63]). All tests were performed using the Tamura-Nei (64) model of nucleotide substitution. For all tests, a random distribution using 1,000 permutations was constructed to assess significance.

### Variable-region analysis.

Nucleotide sequences from the five variable regions (V1 to V5) were extracted from the raw reads for each study participant/tissue/cell. For two samples that contained >90,000 reads, a subset of reads (50%) was randomly sampled. The reads were translated to amino acids, and reads that contained a stop codon were filtered out. Length and charge were determined for all reads by using the variable-region characteristics tool at the Los Alamos HIV-1 Database (https://www.hiv.lanl.gov/content/sequence/VAR_REG_CHAR/index.html). Predicted tropism was determined using the PSSM tool (https://indra.mullins.microbiol.washington.edu/webpssm/). Alignments of the HQCS10 are available at https://github.com/Bioinfoexperts/pacbio.

### Data availability.

Raw sequence data are available in the NCBI Short Read Archive under BioProject no. PRJNA860970.
