# Supplementary material for: Distinct HIV-1 Population Structure across Meningeal and Peripheral T Cells and Macrophage Lineage Cells
Source: Microbiol Spectr. 2022 Sep 29;10(5):e02508-22. doi: 10.1128/spectrum.02508-22 (PMC9602438; doi:10.1128/spectrum.02508-22)
Supplement: Supplemental file 1 — Supplemental material. Download spectrum.02508-22-s0001.pdf, PDF file, 0.3 MB [file spectrum.02508-22-s0001.pdf]

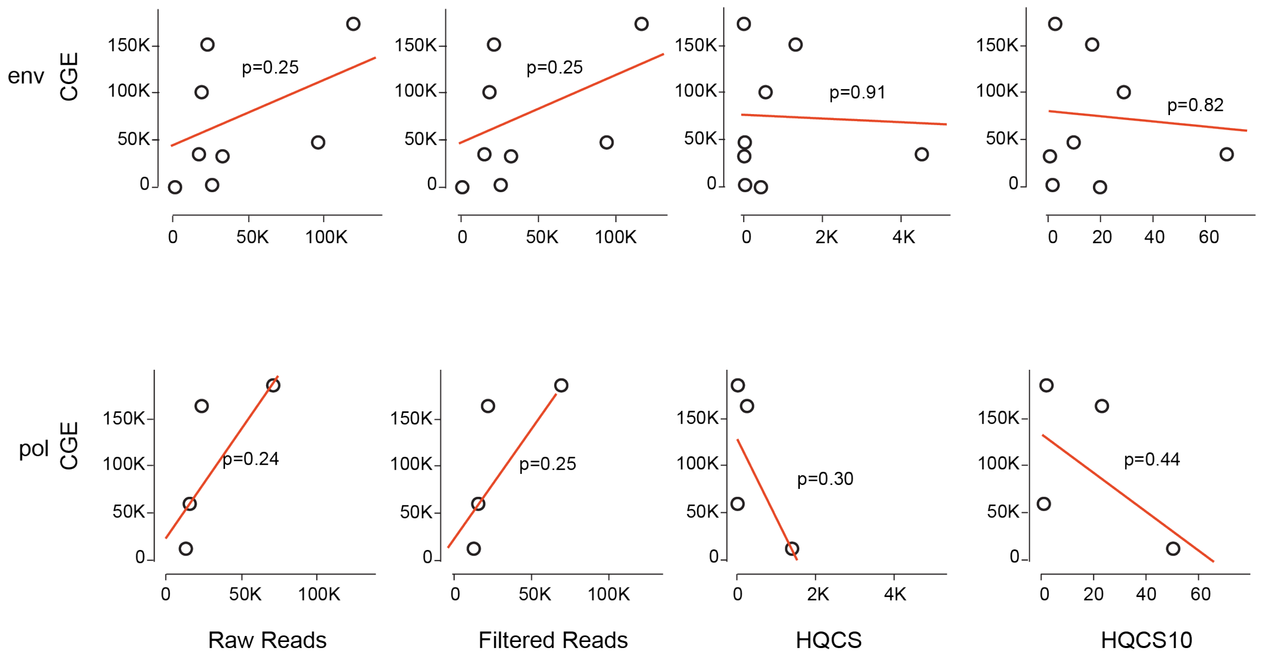

Supplemental Figure 1. Scatterplot of the estimated cell genome equivalent (CGE, y-axis) versus the number of raw/filtered reads and HQCS/HQCS10 (x-axis) for both *env* (top) and *pol* (bottom). The red line is the linear regression, with the significance shown next to the line.

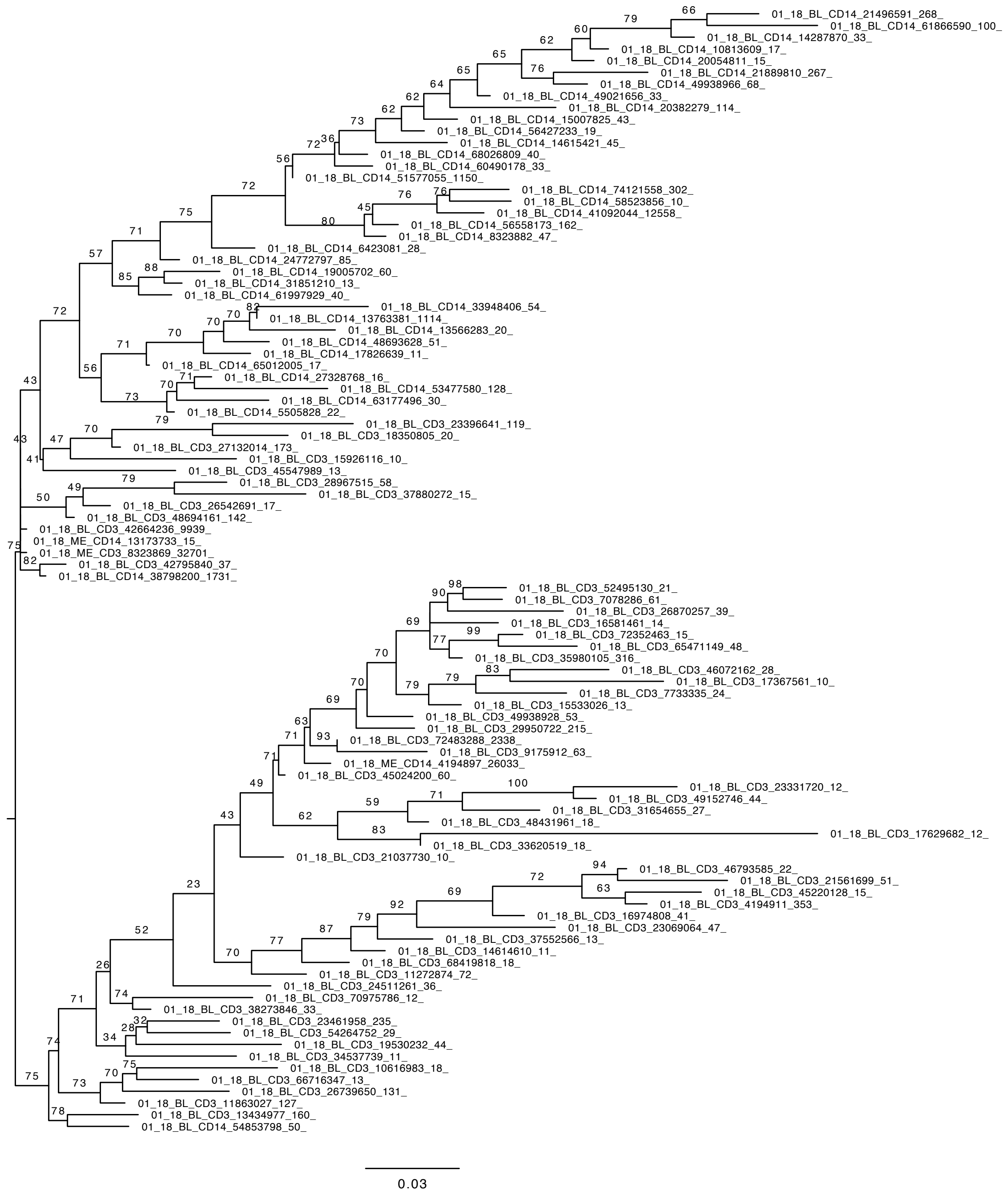

Supplemental Figure 2. Maximum likelihood phylogeny of *env* HQCS10 variants for participant 01-18. Branches are scaled in substitutions per site according to the bar at the bottom. Sequence names indicate the participant (01_18), tissue (BL = blood, ME = meninges) and cell (CD3/CD14) of origin, the 8-digit clone number, and the number of reads represented by each variant. Bootstrap values for each branch are shown.

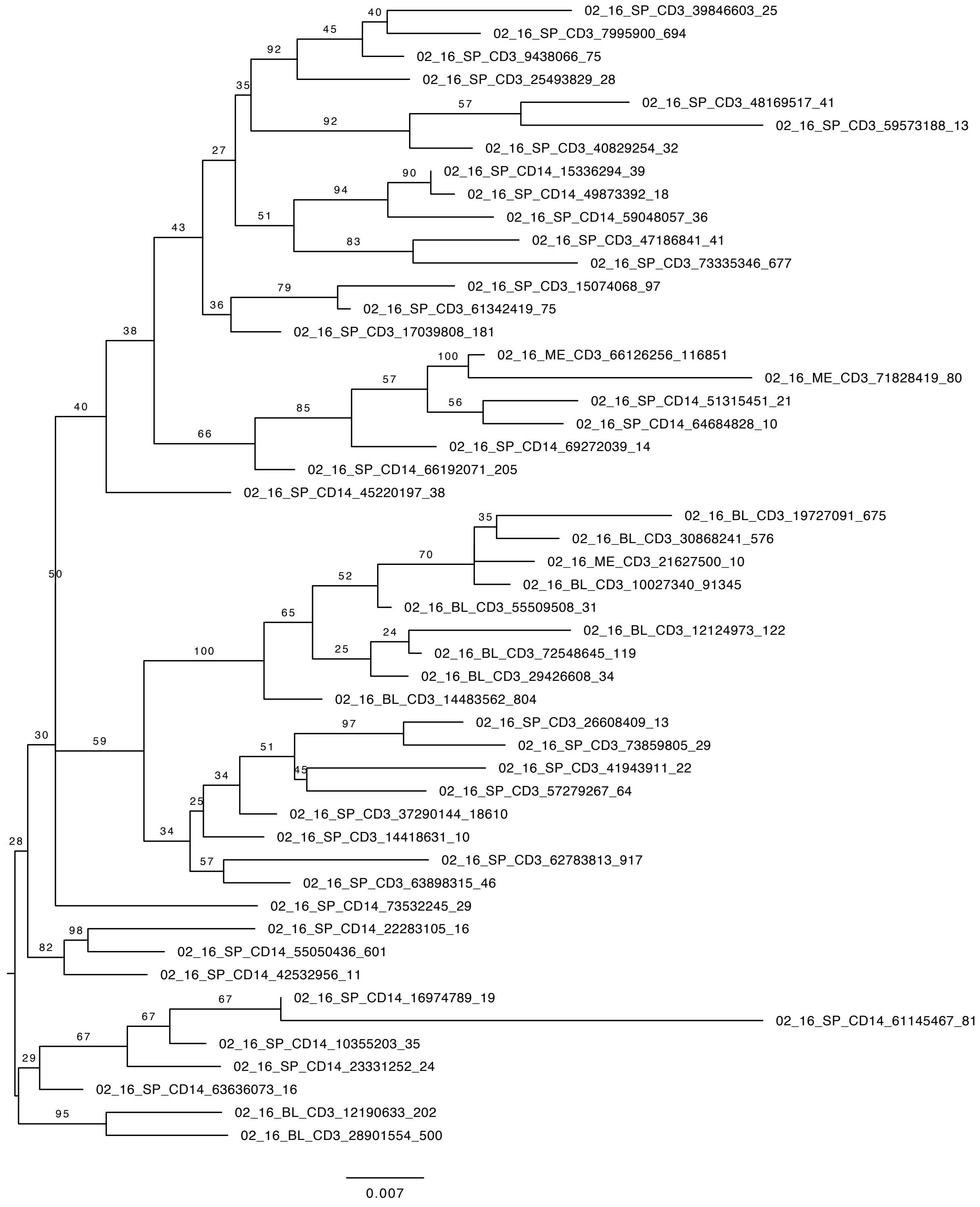

Supplemental Figure 3. Maximum likelihood phylogeny of *env* HQCS10 variants for participant 02-16. Branches are scaled in substitutions per site according to the bar at the bottom. Sequence names indicate the participant (01_18), tissue (BL = blood, ME = meninges) and cell (CD3/CD14) of origin, the 8-digit clone number, and the number of reads represented by each variant. Bootstrap values for each branch are shown.

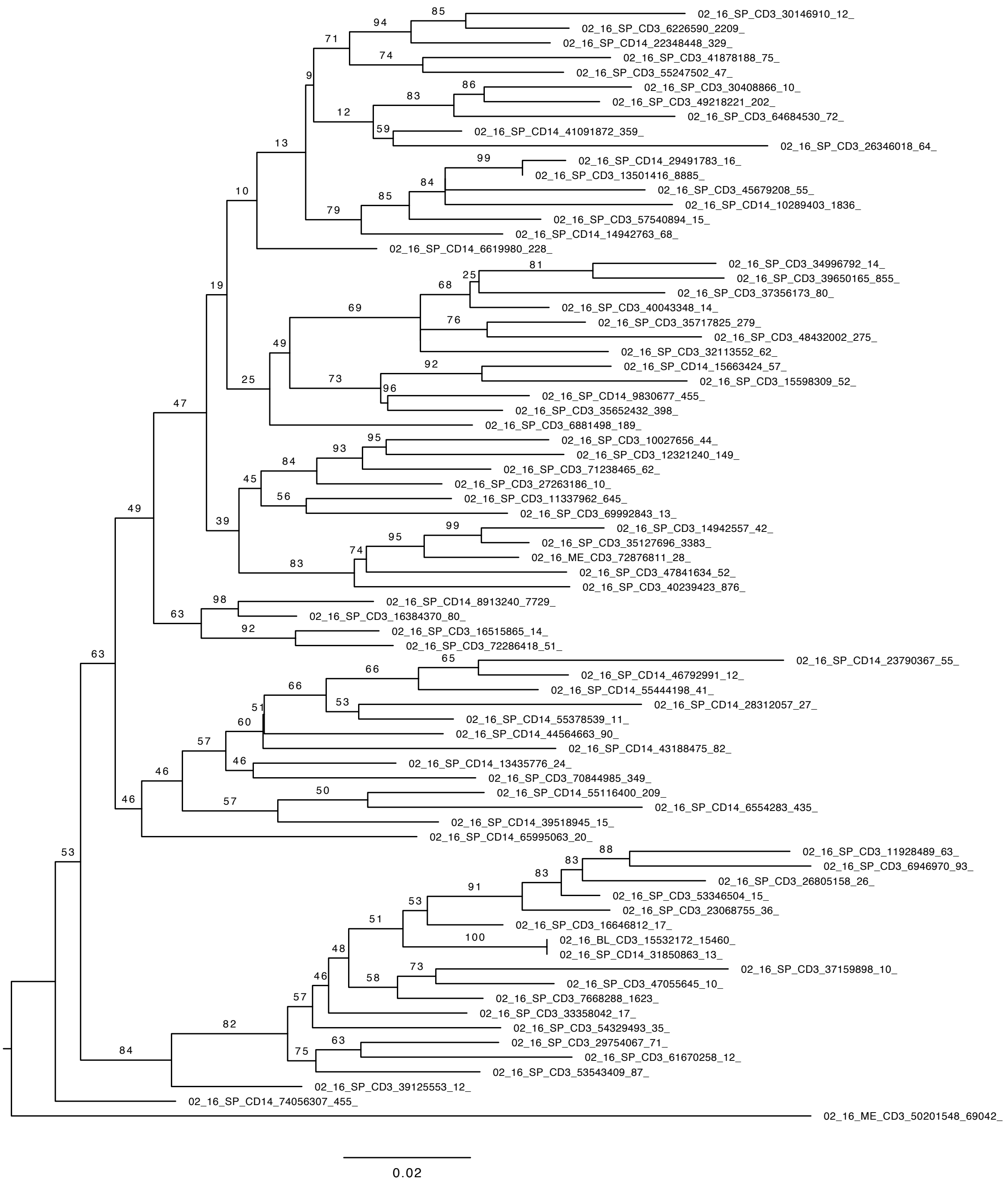

Supplemental Figure 4. Maximum likelihood phylogeny of *pol* HQCS10 variants for participant 02-16. Branches are scaled in substitutions per site according to the bar at the bottom. Sequence names indicate the participant (01_18), tissue (BL = blood, ME = meninges) and cell (CD3/CD14) of origin, the 8-digit clone number, and the number of reads represented by each variant. Bootstrap values for each branch are shown.

01-18 V1V2

```
V1V2_0118_BL_CD14_12749 CTDLKNV----STSDNDTRRNIEDLGMKNCSFKVTTSIRNKMQKEYAAFYKLDLVPIDNDTSYTLISC
V1V2_0118_BL_CD14_2372  CTDLKNVTNTNNTSDNNTRGNIEELEMKNCSFNVTTSIRNKMQKEYAVFYKLDIVPIDNDTSYTLISC
V1V2_0118_BL_CD14_140   CTDLKNV----STSDNDTRRNIEDLGMKNCSFKVTTSIRNKMQKEYAVFYKLDIVPIDNDTSYTLISC
V1V2_0118_BL_CD14_175   CTDLKNVTNTNNTSDNNTRGNIEELEMKNCSFNVTTSIRNKMQKEYAAFYKLDLVPIDNDTSYTLISC
V1V2_0118_BL_CD14_205   CTDLKNV----NTSDNNTRRNIEELGMKNCSFNVTTSIRNKMQKEYAAFYKLDLVPIDNDTSYTLISC
V1V2_0118_BL_CD14_295   CTDLKNV----STSDNDTRRNIEDLGMKNCSFKVTTSIRNKMQKEYAAFYKLDIVPIDNDTSYTLISC

V1V2_0118_BL_CD3_8562   CTDLKNVTNTNNTSDNNTRGNIEELEMKNCSFNVTTSIRNKMQKEYAVFYKLDIVPIDNDTSYTLISC
V1V2_0118_BL_CD3_1021   CTDLKNVT-ANNTSNSITRGNIEELEMKNCSFNVTASIRNKMQKEHAVFYSLDIVPIDN-TSYTLRNC
V1V2_0118_BL_CD3_111    CTDLKNVTTANNTSDNTTRGNIGELEMKNCSFNVTASIRNKMQKEHAVFYSLDIVPIDN-TSYTLRNC
V1V2_0118_BL_CD3_115    CTDLKNVTTANNISDSTTRGNIGELEMKNCSFNVTASIRNKMQKEHAVFYSLDIVPIDN-TSYTLRNC
V1V2_0118_BL_CD3_140    CTDLNNVTTANNTSNNTTRGNIGELEMKNCSFNVTASIRNKMQKEHAVFYSLDIVPIDN-TSYTLRNC
V1V2_0118_BL_CD3_612    CTDLKNVT---NTNDTSNRENIKELEMKNCSFNVTTSIRNKMQKEYALFYKLDIVPIDN-TSYTLINC

V1V2_0118_ME_CD14_25283 CTDLKNVTTANNTGDSTTRGNIGELEMKNCSFNVTASIRNKMQKEHAVFYSLDIVPIDN-TSYTLRNC

V1V2_0118_ME_CD3_32088  CTDLKNVTNTNNTSDNNTRGNIEELEMKNCSFNVTTSIRNKMQKEYAVFYKLDIVPIDNDTSYTLISC
```

01-18 V3

```
V3_0118_BL_CD14_18820   CSRPSNNTRKSIRIGPGSAFFTTGEITGDIRQAHC

V3_0118_BL_CD3_10777    CSRPSNNTRKSIRIGPGSAFFTTGEITGDIRQAHC
V3_0118_BL_CD3_2874     CSRPSNNTRKSIRIGPGSAFYATGEVIGDIRQAHC
V3_0118_BL_CD3_227      CSRPSNNTRKSIRIGPGSAFFTTGEIIGDIRQAHC

V3_0118_ME_CD14_25793   CSRPSNNTRKSIRIGPGSAFYATGEVIGDIRQAHC

V3_0118_ME_CD3_32304    CSRPSNNTRKSIRIGPGSAFFTTGEITGDIRQAHC
```

01-18 V4

```
V4_0118_BL_CD14_12251   CNTTKLFNSTWQSNSTWGNNTGGNETITLPC
V4_0118_BL_CD14_2383    CNTTQLFNSTWLSNGTWGNGTEG--NITLPC

V4_0118_BL_CD3_8587     CNTTQLFNSTWLSNGTWGNGTEG--NITLPC
V4_0118_BL_CD3_2538     CNTTKLFNSTWPSNGTWGNGTEGNETITLPC
V4_0118_BL_CD3_339      CNTTQLFNSTWLSNGTWGNGTEGNETITLPC
V4_0118_BL_CD3_154      CNTTKLFNSTWLSNGTWGNGTEGNETITLPC
V4_0118_BL_CD3_197      CNTTKLFNSTWLSNGTWGNDTEGNETITLPC

V4_0118_ME_CD14_25869   CNTTKLFNSTWPSNGTWGNGTEGNETITLPC

V4_0118_ME_CD3_32488    CNTTQLFNSTWLSNGTWGNGTEG--NITLPC
```

01-18 V5

```
V5_0118_BL_CD14_19077   GYNAS--NETFRP

V5_0118_BL_CD3_10944    GYNAS--NETFRP
V5_0118_BL_CD3_1699     GYNVSNDNETFRP
V5_0118_BL_CD3_221      GYNVS-IHETFRP
V5_0118_BL_CD3_444      GYNVSNNNETFRP
V5_0118_BL_CD3_610      GYNVS--NETFRP

V5_0118_ME_CD14_26017   GYNVSDKNETFRP

V5_0118_ME_CD3_32661    GYNAS--NETFRP
```

02-16 V1V2

```
V1V2_0216_BL_CD3_44179  CTDNLTNVTVGNETRGEIKNCSFNITTSIRDKVQKEYALFYKLDVVPIDDDNTVNNTNITHYRLINC
V1V2_0216_BL_CD3_295    CTDNLTNVTVGNETRGEIKNCSFNITTSIRDKVQKEYALFYKLDVVPIDDDNNANNTNDTHYRLINC
V1V2_0216_BL_CD3_388    CTDNLTNVTVGNETKGEIKNCSFNITTSIRDKVQREYALFYKLDVVPIDDDDTVNNTNITHYRLINC
V1V2_0216_BL_CD3_175    CTDNLTNVTVGNETKGEIKNCSFNITTSIRDKRQREYALFYKLDVVPIDDDDNANNTNDTHYRLINC
V1V2_0216_BL_CD3_290    CTDNLTNVTVGNETRGEIKNCSFNITTSIRDKRQKEYALFYKLDVVPIDDDNNANNTNDTHYRLINC

V1V2_0216_ME_CD3_34882  CTDNLTNVTVGNETRGEIKNCSFNITTSIRDKRQREYALFYKLDVVPIDDDNNANNTNDTYYRLINC

V1V2_0216_SP_CD14_584   CTDNLTNVTVGNETKGEIKNCSFNITTSIRDKVQREYALFYKLDVVPIDDDNNANNTNDTHYRLINC
V1V2_0216_SP_CD14_124   CTDNLTNVTVGNETRGEIKNCSFNITTSIRDKRQKEYALFYKLDVVPIDDDNNANNTNDTHYRLINC
V1V2_0216_SP_CD14_145   CTDNLTNVTVGNETKGEIKNCSFNITTSIRDKVQKEYALFYKLDVVPIDDDNNANNTNDTHYRLINC
V1V2_0216_SP_CD14_160   CTDNLTNVTVGNETRGEIKNCSFNITTSIRDKRQREYALFYKLDVVPIDDDNNANNTNNTYYRLINC

V1V2_0216_SP_CD3_15841  CTDNLTNVTVGNETKGEIKNCSFNITTSIRDKVQKEYALFYKLDVVPIDDDNNANNTNDTHYRLINC
V1V2_0216_SP_CD3_1087   CTDNLTNVTVDNETQGEIKNCSFNITTNIRDKVQREYALFYKLDVVPIDEDNKDNITNSTHYRLINC
V1V2_0216_SP_CD3_655    CTDTLTNVTVGNETKGEIKNCSFNITTSIRDKVRKEYALFYKLDVVPIDDDNKDNSTNGTHYRLINC
V1V2_0216_SP_CD3_449    CTDNLTNVTVDNETQGEIKNCSFNITTSIRDKVQKEYALFYKLDVVPIDDDNNDNNTNDTHYRLINC
V1V2_0216_SP_CD3_303    CTDTLTNVTVGNETKGEIKNCSFNITTSIRDKVQKEYALFYKLDVVPIDDDNNANNTNDTHYRLINC
V1V2_0216_SP_CD3_106    CTDNLTNVTVGNETKGEIKNCSFNITTSIRDKVQKEYALFYKLDVVPIDDDNKANSTNDTHYRLINC
V1V2_0216_SP_CD3_123    CTDNLTNVTVDNETKGEIKNCSFNITTNIRDKVQREYALFYKLDVVPIDDDNKANITNDTHYRLINC
V1V2_0216_SP_CD3_136    CTDNLTNVTVGNETQGEIKNCSFNITTSIRDKVQKEYALFYKLDVVPIDDDNNDNNTNDTHYRLINC
V1V2_0216_SP_CD3_141    CTDTLTNVTVGNETKGEIKNCSFNITTSIRDKVQEEYALFYKLDVVPIDDDNNDNNTNDTHYRLINC
V1V2_0216_SP_CD3_147    CTDNLTNVTVGNETKGEIKNCSFNITTSIRDKVQEEYALFYKLDVVPIDDDNKANSTNDTHYRLINC
V1V2_0216_SP_CD3_170    CTDTLTNVTVGNETKGEIKNCSFNITTSIRDKVQKEYALFYKLDVVPIDDDNNDNNTNDTHYRLINC
V1V2_0216_SP_CD3_183    CTDNLTNVTVDNETKGEIKNCSFNITTNIRDKVQREYALFYKLDVVPIDDDNKANITNSTHYRLINC
V1V2_0216_SP_CD3_202    CTDNLTNVTVGNETKGEIKNCSFNITTSIRDKVQKEYALFYKLDVVPIDDDNNDNNTNDTHYRLINC
V1V2_0216_SP_CD3_220    CTDNLTNVTVDNETQGEIKNCSFNITTSIRDKVQKEYALFYKLDVVPIDDDNNANNTNDTHYRLINC
```

02-16 V3

```
V3_0216_BL_CD3_47175    CTRPNNNTRRGIHMGPGAFYVTGEIIGDIRQAHC

V3_0216_ME_CD3_35120    CTRPNNNTRRGIHMGPGAFYVTGEIIGDIRQAHC

V3_0216_SP_CD14_1290    CTRPNNNTRRGIHMGPGAFYVTGEIIGDIRQAHC
V3_0216_SP_CD14_428     CTRPNNNTRKGIHMGPGAFYVTGEIIGDIRQAHC

V3_0216_SP_CD3_22908    CTRPNNNTRRGIHMGPGAFYVTGEIIGDIRQAHC
```

02-16 V4

```
V4_0216_BL_CD3_46312    CNTSQLFNSTWYANFNSTWYANGTGESNRTDTNITLPC
V4_0216_BL_CD3_241      CNTSRLFNSTWYT--------NGTGESNRTDTNITLPC

V4_0216_ME_CD3_35214    CNTSQLFNSTWYA--NSTWSVNGTGESNKTDTNITLPC

V4_0216_SP_CD14_114     CNTTQLFNSTWNF--------NGTGESDRNDTNITLPC
V4_0216_SP_CD14_151     CNTSQLFNSTWYV--------NGTGESNRTDTNITLPC
V4_0216_SP_CD14_154     CNTTQLFNSTWNF--------NGTEGSNKTDTNITLPC
V4_0216_SP_CD14_185     CNTSQLFNSTWYA--------NSTGESNKTDTNITLPC
V4_0216_SP_CD14_642     CNTTQLFNSTWHF--------NGTGESNRTDTNITLPC

V4_0216_SP_CD3_19131    CNTSQLFNSTWYA--NSTWYANGTGESNRTDTNITLPC
V4_0216_SP_CD3_120      CNTSQLFNSTWYA--NSTWYVNGTGESNRTDTNITLPC
V4_0216_SP_CD3_1436     CNTTQLFNSTWNF--------NDTEESNRTDTNITLPC
V4_0216_SP_CD3_903      CNTSQLFNSTWYT--NSTWNFNSTEGSNRTDTNITLPC
```

02-16 V5

```
V5_0216_BL_CD3_46291    GI---NRTNETFRP
V5_0216_BL_CD3_1050     GT---NRTNETFRP
```

```
V5_0216_ME_CD3_35276    GN-ESQSTNETFRP

V5_0216_SP_CD14_115     GNNGTENETEIFRP
V5_0216_SP_CD14_165     GN-ESQSKNETFRP
V5_0216_SP_CD14_191     GI---NRTNETFRP
V5_0216_SP_CD14_228     GN-ESQSTNETFRP
V5_0216_SP_CD14_645     GN--VSRDNETFRP

V5_0216_SP_CD3_19307    GI---NRTNETFRP
V5_0216_SP_CD3_1024     GN-ESQSTNETFRP
V5_0216_SP_CD3_1309     GN-ESRSNNETFRP
V5_0216_SP_CD3_121      GI---NRSNETFRP
V5_0216_SP_CD3_119      GN-ESQSNNETFRP
V5_0216_SP_CD3_128      GN---ESTNETFRP
V5_0216_SP_CD3_130      GN--DRS-NKTFRP
V5_0216_SP_CD3_166      GN-ESRSTNETFRP
```

Supplemental Figure 5. Majority variant amino acid sequences for V1V2 and V4 regions. Sequences that represent >100 reads at 100% identity is shown for each tissue/cell. The position of the residues relative to the longest sequence is shown for each alignment. Sequence names indicate the participant (01_18), tissue (BL = blood, ME = meninges) and cell (CD3/CD14) of origin, the 8-digit clone number, and the number of reads represented by each read.
